# Molecular Design Based on Donor-Weak Donor Scaffold for Blue Thermally-Activated Delayed Fluorescence Designed by Combinatorial DFT Calculations

**DOI:** 10.3389/fchem.2020.00403

**Published:** 2020-05-06

**Authors:** Youichi Tsuchiya, Keita Tsuji, Ko Inada, Fatima Bencheikh, Yan Geng, H. Shaun Kwak, Thomas J. L. Mustard, Mathew D. Halls, Hajime Nakanotani, Chihaya Adachi

**Affiliations:** ^1^Center for Organic Photonics and Electronics Research (OPERA), Kyushu University, Fukuoka, Japan; ^2^Japan Science and Technology Agency (JST), ERATO, Adachi Molecular Exciton Engineering Project, Fukuoka, Japan; ^3^Department of Chemistry and Biochemistry, Kyushu University, Fukuoka, Japan; ^4^Schrödinger Inc, San Diego, CA, United States; ^5^International Institute for Carbon Neutral Energy Research (WPI-I2CNER), Kyushu University, Fukuoka, Japan

**Keywords:** TADF, homo-junction design, combinatorial DFT calculation, photo-physics, four-state rate equations

## Abstract

Quantum chemical calculations are necessary to develop advanced emitter materials showing thermally-activated delayed fluorescence (TADF) for organic light-emitting diodes (OLEDs). However, calculation costs become problematic when more accurate functionals were used, therefore it is judicious to use a multimethod approach for efficiency. Here we employed combinatorial chemistry *in silico* to develop the deep blue TADF materials with a new concept of homo-junction design. The homo-junction materials containing TADF candidates designed by calculation were synthesized and analyzed. We found that these materials showed the emission from charge transfer (CT) state, and the clear delayed emission was provided in solid state. Because the homo-junction TADF materials showed three exponential decayed emission in solid state, we employed novel four-state kinetic analysis.

## Introduction

TADF materials have received significant attention for application in high efficiency OLEDs. This is because TADF materials can realize nearly 100% internal quantum efficiency of electroluminescence by harvesting all electrically generated singlet and triplet excitons as prompt and delayed emission. The effective upconversion of excitons from a triplet excited-state (T_1_) to a singlet excited-state (S_1_) is possible because of the small energy splitting between the S_1_ and T_1_ states (Δ*E*_ST_), resulting in nearly 100% reverse intersystem crossing (RISC) efficiency (Uoyama et al., 2012). While the TADF phenomenon has been known as E-type delayed fluorescence since the 1940s (Lewice et al., 1941), no comprehensive molecular design aiming to realize high efficiency TADF has been reported. In 2012, our group reported high efficiency TADF materials by molecular design with electron donor (D) and acceptor (A) units separated by a distance controlled by a linkage/spacer unit (Uoyama et al., 2012). Because Δ*E*_ST_ is theoretically proportional to the exchange integral *J* (Yersin, 2018), then

(1) $$\begin{array}{r} \Delta E_{ST}=E_{S}-E_{T}=2J \end{array}$$

*J* depends on the electron density overlap between the highest occupied molecular orbital (HOMO) and lowest unoccupied molecular orbital (LUMO), i.e.,

(2) $$J=\iint \phi_{H}(r_{1})\phi_{L}(r_{2})\frac{1}{\left|r_{2}-r_{1}\right|}\phi_{H}(r_{2})\phi_{L}(r_{1})dr_{1}dr_{2}$$

where ϕ_*H*_ and ϕ_*L*_ are the spatial distributions of the HOMO and LUMO, respectively, and **r**_1_ and **r**_2_ are position vectors. Thus, it follows that reducing the overlap integral between the HOMO and LUMO decreases the *J* and Δ*E*_ST_. The oscillator strength *f*, which is an index for the light emission intensity, is proportional to the square of the transition dipole moment *Q*.

(3) $$\begin{array}{r} f\propto |Q{|}^{2} \end{array}$$

Here, the magnitude of *Q* also depends on the orbital overlap between the HOMO and LUMO, i.e.,

(4) $$Q=\iint \phi_{H}(r_{1})\phi_{L}(r_{2})\left|r_{2}-r_{1}\right|\phi_{H}(r_{2})\phi_{L}(r_{1})dr_{1}dr_{2}$$

These equations indicate that a well-tuned partial orbital overlap between the HOMO and LUMO is a requisite condition for obtaining both a small Δ*E*_ST_ and high emissivity. The molecular design of a D-A pair combined with π-linkers providing a large dihedral angle or insulating σ-spacers can separate the HOMO and LUMO with a small orbital overlap (Tanaka et al., 2012; Geng et al., 2017). Based on this concept, a wide variety of D-A “hetero-junction” type TADF materials were developed by combining D units that are easily oxidized and A units that are easily reduced, resulting in highly efficient TADF materials (Wong and Zysman-Colman, 2017; Yang et al., 2017; Bui et al., 2018; Liu et al., 2018). However, this D-A architecture largely limits the composite selection to triphenylamines and carbazoles derivatives as donors, and triazines, cyanobenzenes, oxadiazoles, sulphonyls, and carbonyls derivatives as acceptors. Such limitation makes it difficult to expand the TADF scaffold. In particular, there is strong demand for blue TADF emitters based on new molecular structures. General consensus has been that the D-A architecture is mandatory to obtain TADF characteristics. Basically, D and A moieties are categorized by the major properties of each unit with considering its substituents (Hansch et al., 1991). However, we recognized that separation of the HOMO and LUMO, that is, a small Δ*E*_ST_, can theoretically be obtained even by strong donor-weak donor (_S_D-_W_D) and strong acceptor-weak acceptor (_S_A-_W_A) combinations. Because the electron donating and accepting ability is relative between two moieties. Thus, even the homo combination of _S_D-_W_D and _S_A-_W_A should theoretically provide TADF characteristics when the HOMO and LUMO are separated and the material have a small Δ*E*_ST_. The homo-junction molecular design approach will alleviate the limitation of unit selection for novel blue TADF materials. In this study, we examined the design of TADF materials based on a homo-junction of _S_D-_W_D, particularly aimed for blue TADF emitters.

## Results and Discussions

### Molecular Design Through Combinatorial DFT Calculations

**Figure 1:**
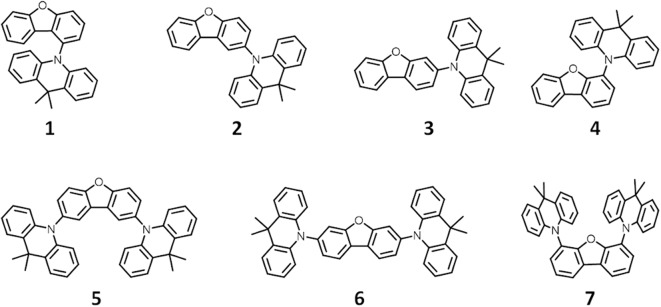
Chemical structures of TADF candidates with homo-junction design.

In this work, density functional theory (DFT) analysis was used to identify synthetic motifs for promising blue TADF candidates. First, we estimated Δ*E*_ST_ values for four molecules with a homo-junction design by DFT calculations with a B3LYP/6-31+g^*^ level of theory. The synthesized compounds showed very large Δ*E*_ST_ values compared with the calculated Δ*E*_ST_ values (Table S1). These large differences were based on the underestimation of exciton energy by the standard functional, B3LYP (Dreuw and Head-Gordon, 2004). Δ*E*_ST_ values estimated using the LC-ωPBE functional with long-range correction using a tuned ω value showed good agreement with the experimental values (Sun et al., 2015). Most of compounds calculated in homo-junction design showed a large Δ*E*_ST_ value in LC-ωPBE level while the value is small in B3LYP level. Ideally, we would have employed the tuned ω LC-ωPBE functional to evaluate the homo-junction molecules. However, DFT calculations with the tuned ω LC-ωPBE functional is excessively time-consuming. Therefore, we used the combinatorial DFT calculation to discover/locate blue TADF candidates with the homo-junction design using the MacroModel, Jaguar and Gaussian16 software packages. The 87 donor units used to construct TADF molecules were analyzed, and the HOMO, LUMO, S_1_ and T_1_ energy levels were estimated by DFT calculation with the B3LYP/6-31+g^*^ level of theory. Figure S1 shows the correlation between the HOMO and LUMO for these composite units. For example, dibenzofuran (DBF, HOMO = −0.23 eV), carbazole (HOMO = −0.21 eV) and 9,9-dimethylacridane (DMAc, HOMO = −0.19 eV) were located in the weak, middle and strong electron donating groups, respectively. Generated _S_D-_W_D combinations using these donor units were screened by the DFT calculation with different functional levels of theory (B3LYP, M06-2X, and LC-ωPBE). All donor fragments with one, two or more modifiable locations were then modified with all donors with only one modifiable location. This generated a library of 2618 candidate _S_D-_W_D TADF molecules. Candidates were then verified by further DFT calculations with the LC-ωPBE/6-31+g^*^ level of theory. From the obtained TADF candidates, we chose the DMAc-DBF combinations listed in Figure 1 and Table S2 as the final blue TADF candidates for the homo-junction design.

### Photophysical Properties of Homo-Junction Materials

**Figure 2:**
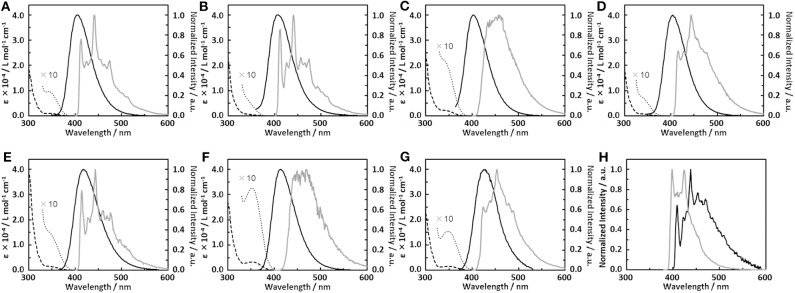
**(A–G)** UV absorption (dashed line), fluorescence (solid black line) and phosphorescence (gray line) spectra of dimethylacridine-dibenzofurane combinations; **(A) 1**, **(B) 2**, **(C) 3**, **(D) 4**, **(E) 5**, **(F) 6**, **(G) 7**; 10 times enlarged absorption spectrum around 350 nm (dotted line). **(H)** Phosphorescence spectra of dibenzofurane (black line) and 9,9-dimethyl-10-phenylacridine (**Ph-DMAc**, gray line).

We therefore synthesized seven _S_D-_W_D materials shown in Figure 1, which included the molecules suggested by the results of the calculation. All compounds with the homo-junction design showed good HOMO-LUMO separation similar to that for the conventional D-A design (Table S3). The natural transition orbitals (NTO) for the lowest singlet excitation of **1**–**7** showed good agreement with their HOMO and LUMO distributions. The Figure 2 shows ultraviolet (UV) absorption, fluorescence and phosphorescence spectra of **1**–**7** in toluene solution (1.0 × 10^−5^ mol L^−1^). By Gauss curve fitting of the absorption spectra (Figure S2), the molar absorption coefficients of the CT absorptions (around 350 nm) for **1**, **2**, **3**, **4**, **5**, **6**, and **7** were estimated to be 1019, 501, 1793, 680, 1477, 3168, and 1522 L mol^−1^ cm^−1^, respectively (Table 1). While this clearly indicated the presence of CT transitions in the _S_D-_W_D molecules, the experimentally estimated oscillator strength *f* of the CT absorption was quite small at < 0.03 (Hirata et al., 2015). All compounds showed emission in the deep blue region (FL_max_ values were shown at 400–430 nm) with large full width at half maximum (FWHM) values of around 70 nm and non-vibronic structure, indicating that the fluorescence originated from the CT excited state. The PLQYs of all compounds were low, reflecting the weak oscillator strength in toluene solution. The trend in radiative decay rate constant ($k_{r}^{S}$) estimated from the absorption and fluorescence spectra showed good agreement with the rate constants from emission decay measurements. Larger *f* and *Q* values of **3** and **6**, rather than other mono-substituted series, means the modification on 3 and 7 positions of DBF have larger orbital overlap between HOMO on DMAc and LUMO on DBF than others. On the other hand, **2** and **4** have similar *f*, *Q* values, and other photophysical properties. Therefore, the photophysical properties would not be affected by the donor modification of 2 (8) and 4 (6) positions on DBF. Furthermore, the photochemical properties of compounds **2**, **4**, **5**, and **7** can be directly compared. The modification of two strong donors showed red-shifted emission, meaning reduction of the S_1_ energy level which is also observed in conventional D-A type TADF molecules in generally (Wong and Zysman-Colman, 2017; Yang et al., 2017; Bui et al., 2018; Liu et al., 2018). The S_1_ energy of **7** showed a larger red-shift than **5** (0.09 and 0.14 eV for **5** and **7** from **2** and **4**, respectively), however, the T_1_ energy shift scarcely had any different because of the local excited (LE) state as described hereinbelow. The red-shifted CT emission between the same interacting groups meaning an enhancement of interaction strength between D-A. As a result, 4,6-modification of DBF provided smaller Δ*E*_ST_ values than 2,8-modification of DBF (Figure S3). The smaller Δ*E*_ST_ should mean the material has small orbital overlap as shown by Equations 1 and 2, however, we could not find the large difference in *f* values.

**Table 1:** Photophysical values of compounds **1**–**7** in toluene.

	**1**	**2**	**3**	**4**	**5**	**6**	**7**
$\lambda_{\text{abs}}^{\text{CT}}$ (nm)^a^	345	341	345	340	342	356	349
ε^CT^ (L mol^−1^ cm^−1^)^a^	1019	501	1793	680	1477	3168	1522
*f* ^[b]^	0.010	0.004	0.014	0.005	0.016	0.026	0.015
*Q* ^[b]^	0.932	0.612	1.122	0.686	1.197	1.526	1.170
$k_{\text{r}}^{\text{S}}$ (10^7^ s^−1^)^b^	1.17	0.54	1.82	0.74	1.73	2.92	1.56
PLQY^c^	0.047	0.057	0.182	0.040	0.053	0.175	0.063
FL_max_	406	405	402	404	418	415	428
S_1_ (eV)^d^	3.32	3.33	3.35	3.34	3.24	3.25	3.20
T_1_ (eV)	3.01^e^	3.02^e^	2.98^d^	3.00^d^	3.01^e^	2.92^d^	3.01^d^
ΔE_ST_ (eV)	0.31	0.31	0.38	0.34	0.23	0.33	0.19
τ_PL_ (ns)^c^	3.37	6.34	2.32	3.89	8.26	4.46	7.04
τ_DE_ (μs)^c^	0.45	0.27	–	0.25	0.26	–	0.30
$k_{\text{r}}^{\text{S}}$ (10^7^ s^−1^)^c^	1.34	0.85	7.72	1.02	0.63	3.88	0.84
*k*_ISC_ (10^8^ s^−1^)^c^	2.84	1.42	3.54	2.47	1.14	1.85	1.33
*k*_RISC_ (10^4^ s^−1^)^c^	9.21	3.62	–	4.98	4.29	–	4.37
$k_{\text{nr}}^{\text{T}}$ (10^6^ s^−1^)^c^	2.20	3.71	–	4.08	3.86	–	3.29

a *Results from Gauss curve fitting (see Figure S2)*.

b *Estimated using equations reported in literature (see Supporting Information)*.

c *Inert gas saturated conditions*.

d *Estimated from the onset value of the spectrum*.

e *Estimated from the shortest wavelength peak maximum in the phosphorescence spectrum*.

All materials showed very strong phosphorescence in frozen toluene at 77 K. The PLQYs at 77 K reached nearly 100%, confirming that non-radiative decay from the T_1_ state in **1**–**7** was the main deactivation process at room temperature. The phosphorescence spectral profiles of **1**–**7** showed significant differences with substitution position on the DBF. The vibronic structures based on the DBF were clearly observed by DMAc modification at the 1-, 2- and 4-position of DBF (**1**, **2** and **4**), while 3 showed broad CT emission. The phosphorescence spectra of the di-functionalized compounds were consistent with the respective mono-modified compounds. Overall, the T_1_ states of **1**, **2**, **4**, **5**, and **7** originated from the LE states of the DBFs, and those of **3** and **6** were based on the CT states between the DMAc and DBF units. Four and seven from the emission lifetime measurements in toluene solution, **1**, **2**, **4**, **5**, and **7** showed a very weak long lifetime component under N_2_ saturated conditions (Figure S4). These long lifetime decay components were suppressed under air saturated conditions, so the delayed component was ascribed to TADF.

### Relative Electron Density on Homo-Junction Materials

**Figure 3:**
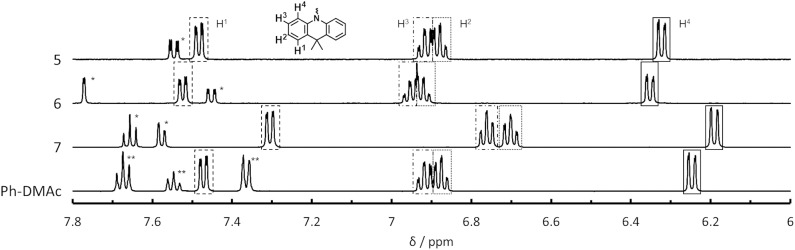
^1^H NMR spectra of **5**, **6**, **7** and Ph-DMAc in THF-*d*_8_ solution; * and ** indicate peaks arising from DBF and Ph groups, respectively.

We found an interesting relationship between the Δ*E*_ST_ values of **5**–**7** and their proton nuclear magnetic resonance (^1^H NMR) spectra. The chemical shift in the NMR spectra indicates the shielding strength of the magnetic field to the atomic nucleus by the electron orbitals. Thus, the NMR spectra allow the relative electron density on each composite unit to be determined (Schaefer and Schneider, 1963). Figure 3 shows ^1^H NMR spectra for the aromatic protons on the DMAc unit for **5**–**7** and for *N*-phenyl-9,9-dimethylacridane (Ph-DMAc) (^1^H NMR data for **1**–**7** including the DBF unit region are shown in Figure S5, and chemical shifts are summarized in Tables S4, S5). The peaks of **5** and **6** showed similar chemical shifts or slightly downfield shifts compared with those of **Ph-DMAc**. Compound **7** showed upfield shifts for all aromatic protons on the DMAc unit. The methyl group on the DMAc unit of **7** showed a strong upfield shift (Δδ = 0.26 ppm) without peak splitting. This upfield shift for **7** compared with that of Ph-DMAc indicates that the DBF unit is an electron donor in the ground state. This may have reflected the resonating effect of the lone electron pair on the DBF oxygen atom. Thus, **7** achieved efficient HOMO-LUMO separation while retaining the conjugation by the electron back donation from DMAc to DBF units at the excited state. Smaller Δ*E*_ST_ values of 0.33, 0.23 and 0.19 eV were obtained for **6**, **5** and **7**, respectively, and this trend well-corresponded to the opposite magnitude of the relative electron density on DMAc; that was **6** < **5** < **7**. The electron donation from DBF (_W_D) to DMAc (_S_D) at the ground state could also be observed from the shift of the HOMO level. Compound **7** showed a shallower HOMO level (−5.73 eV) than those of **5** and **6** (−5.85 and −5.84 eV, respectively), indicating that the electron-rich DMAc group played an important role in achieving the small Δ*E*_ST_ for TADF. The peaks of **4** showed no upfield shift compared to **Ph-DMAc** and were similar to those for other mono-substituted compounds except for **1**. This indicated that the upfield shift of **7** did not originate from the ring current effect by the DBF unit. A clear ring current effect was observed in the NMR spectrum of **1** with the peak splitting of methyl groups on the DMAc units. The mono-substituted **4** showed a larger high magnetic field shift of 6-position proton on the unmodified phenyl ring of DBF instead on DMAc than **2**, **3** and DBF (Figure S5 and Table S5). In addition, a low magnetic field shift for **7** compared with **4** were observed for the comparable proton peak on DBF. The strong electronic interaction found in **7** between DMAc and DBF might induce the energy shift of S_1_ and resulting the smaller Δ*E*_ST_ values.

### Solid State Photophysical Properties With Four State Analysis

**Figure 4:**
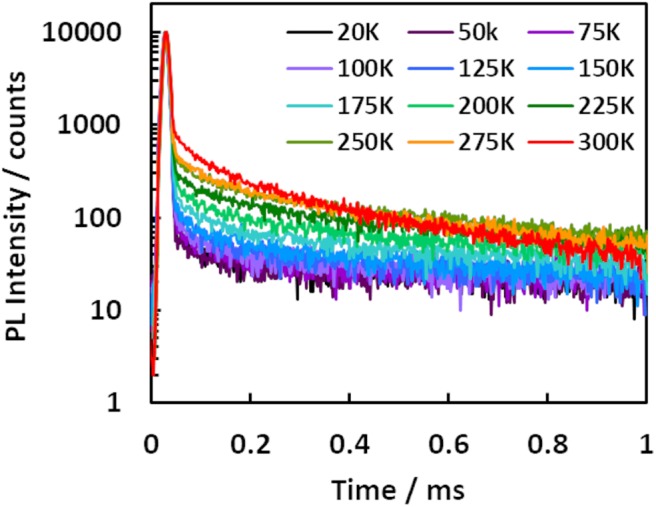
Temperature dependency of transient emission decay of a DPEPO film doped with **7** (6 wt%).

**Figure 5:**
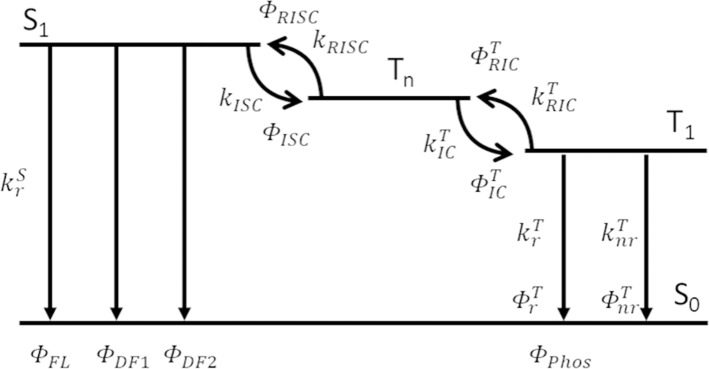
Kinetic analysis model of TADF with four states.

All compounds **1**–**7** showed delayed emission in a bis[2-(diphenylphosphino)phenyl]ether oxide (DPEPO) host (6 wt%), and the delayed components for **1**–**7** were suppressed by decreasing the temperature (Figure 4, Figures S6, S7). Therefore, all delayed emissions for **1**–**7** at room temperature were ascribed to TADF. However, the TADF activity was very small in **3** and **6**, reflecting their solution properties. Because **7** showed clear temperature dependence of the emission decay profile in the DPEPO host (6 wt%), a detailed kinetic analysis was performed. In Figure 4, the long lifetime component clearly increased with increasing temperature, indicating that the delayed emission observed at higher temperatures was based on TADF. Two decay components of τ_*DE*1_ = 0.17 ms and τ_*DE*2_ = 2.20 ms were observed at 300 K in the delayed emission. The spectrum of the longer delayed emission (1–10 ms) gradually blue-shifted with increasing temperature (Figure S8). This indicated that the secondary delayed component was not pure phosphorescence but rather the mixed emission of TADF and phosphorescence, and their ratio gradually changed with increasing temperature. While the reported three-state (S_0_, S_1_, and T_1_) model could not well-explain these three order decays (Masui et al., 2013), the expanded four-state (S_0_, S_1_, T_1_, and T_n_) model could well explain it (Figure 5). Recent reports revealed that the T_n_ state plays an important role for efficient RISC (Etherington et al., 2016; Kobayashi et al., 2017; Noda et al., 2019). These reports revealed the T_n_ state acts as an intermediate state of intersystem crossing (ISC) and RISC processes. Our results could be explained by a similar TADF system with multi-exponential decays. Therefore, we applied the four-state kinetic analysis. In this case, these two delayed components are explained by the decay rates of T_n_ ($k_{1}^{T}=\frac{1}{\tau_{DE1}}$) and T_1_ ($k_{2}^{T}=\frac{1}{\tau_{DE2}}$). When we assumed that there are very small contribution of direct non-radiative decay from both the S_1_ and T_n_ states to S_0_ ($\Phi_{nr}^{S},\;\Phi_{nr}^{Tn}\approx 0$), and very small contribution of direct ISC and RISC between the S_1_ and T_1_ states, we can obtain the rate equations. Thus, $k_{r}^{S}$ and ISC rate (*k*_*ISC*_) of the S_1_ state are written by

(5) $$\begin{array}{r} k_{r}^{S}=k^{S}\Phi_{FL} \end{array}$$

(6) $$\begin{array}{r} k_{ISC}=k^{S}\Phi_{ISC} \end{array}$$

where *k*^*S*^ is the decay rate of the S_1_ exciton ($k^{S}=\frac{1}{\tau_{FL}}$, τ_*FL*_ is the lifetime of the fluorescence component), Φ_*FL*_ is the PL efficiency of the fluorescence component and Φ_*ISC*_ is the ISC efficiency (1 − Φ_*FL*_). Because the T_n_ exciton had only two decay paths, the rate constants for RISC (*k*_*RISC*_) to S_1_ and for exothermic internal conversion ($k_{IC}^{T}$) to T_1_ are written by

(7) $$\begin{array}{r} k_{RISC}=\frac{\Phi_{DE1}}{\Phi_{FL}}\cdot \frac{k^{S}k_{1}^{T}}{k_{ISC}} \end{array}$$

(8) $$\begin{array}{r} k_{IC}^{T}=k_{1}^{T}-\Phi_{FL}k_{RISC} \end{array}$$

where Φ_*DE*1_ is the PL efficiency of the first delayed component (Φ_*DF*1_ in Figure 5). Because the PL efficiency of the second delayed component (Φ_*DE*2_) was the total of the emission from the T_1_ state *via* endothermic reverse internal conversion ($\Phi_{RIC}^{T}$) and radiative decay ($\Phi_{r}^{T}$), i.e., Φ_*DF*2_+Φ_*Phos*_ in Figure 5, their rate constants ($k_{RIC}^{T}$ and $k_{r}^{T}$) are written by

(9) $$\begin{array}{r} \Phi_{RIC}^{T}+\Phi_{r}^{T}=\frac{\Phi_{DE2}}{\Phi_{ISC}\Phi_{IC}^{T}} \end{array}$$

(10) $$\begin{array}{r} k_{RIC}^{T}=k_{2}^{T}\cdot \frac{\Phi_{RIC}^{T}}{\Phi_{RISC}} \end{array}$$

(11) $$\begin{array}{r} k_{r}^{T}=k_{2}^{T}\Phi_{r}^{T} \end{array}$$

where $\Phi_{IC}^{T}$ and Φ_*RISC*_ are the efficiencies of exothermic internal conversion (IC) and RISC, respectively ($\Phi_{IC}^{T}=1-\Phi_{RISC}$). The efficiency of non-radiative decay from T_1_ ($\Phi_{nr}^{T}$) could be estimated from the total PL efficiency (Φ_*PLQY*_), and its rate constant is written by

(12) $$\begin{array}{r} k_{nr}^{T}=k_{2}^{T}\cdot \frac{1-\Phi_{PLQY}}{\Phi_{ISC}\Phi_{IC}^{T}} \end{array}$$

The phosphorescence (Φ_*phos*_) ratio in the third decay component (Φ_*DE*2_) could be estimated from the time dependent spectra. In this case, all rate constants were provided experimentally from Equation 9. There was no difference of the Φ_*PL*_ values (Table 2) at 100 and 300 K, so we can use an assumption of $\Phi_{nr}^{S}$ as 0. Table 2 shows the rate constants for **7** in a DPEPO film (6 wt%). The values of $k_{r}^{S}$ and *k*_*ISC*_ at 300 K showed good agreement with the result in toluene solution. The *k*_*RISC*_ showed an acceptable difference compared to that in the solution state (Table 1, values using an assumption of $\Phi_{r}^{T}$ as 0 for **1**–**7** are provided in Table S6). The four-magnitude-smaller value of $k_{nr}^{T}$ compared with the solution state suggested suppressed molecular vibration in the solid state at 300 K. Because of the strong molecular vibration in solution, most of the T_1_ excitons would have decayed non-radiatively without reverse internal conversion (RIC). Therefore, only a small delayed emission was observed in solution. The IC speed would have been considerably decreased in the solid state when a large configuration difference was necessary between the T_n_ and T_1_ states. That explained why some TADF materials showed an additional long exponential decay in their delayed emission. The rate constants of the endothermic processes (*k*_*RISC*_ and $k_{RIC}^{T}$) were suppressed at low temperature, but the rate constants of the exothermic processes ($k_{r}^{S}$, *k*_*ISC*_ and $k_{IC}^{T}$) were largely unchanged except for $k_{nr}^{T}$. These results clearly indicated that the slow endothermic IC with the large conformation change was detrimental to TADF activity. It is necessary to continue discussion about what is happening in the actual photophysical process. None-the-less, the expanded kinetic analysis should reveal details of the photophysical behavior of TADF in the solid state.

**Table 2:** Observed photophysical values and rate constants of 6 wt% 7: DPEPO film at **(A)** 300 K and **(B)** 100 K.

**(A)**						
**Φ_**PLQY**_**	**τ_FL_ (ns)**	**τ_DE1_ (μs)**	**τ_DE2_ (ms)**	**Φ_**PL**_**	**Φ_**DE1**_**	**Φ_**DE2**_**
0.15	3.4	172.0	2.2	0.036	0.043	0.072
$k_{\text{r}}^{\text{S}}$ **(s**^**−1**^**)**	***k***_**ISC**_ **(s**^**−1**^**)**	***k***_**RISC**_ **(s**^**−1**^**)**	$k_{\text{IC}}^{\text{T}}$ **(s**^**−1**^**)**	$k_{\text{RIC}}^{\text{T}}$ **(s**^**−1**^**)**	$k_{\text{r}}^{\text{T}}$ **(s**^**−1**^**)**	$k_{\text{nr}}^{\text{T}}$ **(s**^**−1**^**)**
1.05 × 10^7^	2.84 × 10^8^	7.15 × 10^3^	5.56 × 10^3^	7.19 × 10^2^	3.58	4.18 × 10^2^
**(B)**						
***Φ***_**PLQY**_	**τ_FL_ (ns)**	**τ_DE1_ (μs)**	**τ_DE2_ (ms)**	***Φ***_**PL**_	***Φ***_**DE1**_	***Φ***_**DE2**_
0.98	4.9	264.4	339.4	0.028	0.005	0.944
$k_{\text{r}}^{\text{S}}$ **(s**^**−1**^**)**	***k***_**ISC**_ **(s**^**−1**^**)**	***k***_**RISC**_ **(s**^**−1**^**)**	$k_{\text{IC}}^{\text{T}}$ **(s**^**−1**^**)**	$k_{\text{RIC}}^{\text{T}}$ **(s**^**−1**^**)**	$k_{\text{r}}^{\text{T}}$ **(s**^**−1**^**)**	$k_{\text{nr}}^{\text{T}}$ **(s**^**−1**^**)**
1.05 × 10^7^	1.98 × 10^8^	6.45 × 10^2^	3.76 × 10^3^	34.8	2.71	0.072

### OLED Derivatives Using Homo-Junction Materials as an Emitter

Finally, we fabricated an OLED with **7** as an emitter (Figure S8) with the device structure of indium tin oxide (ITO)/*N, N*'-di-1-naphthyl-*N, N*'-diphenylbenzidine (α-NPD, 20 nm)/4,4',4“-tris (carbazol-9-yl)-triphenylamine (TCTA, 20 nm)/3-bis(9-carbazolyl) benzene (mCP, 10 nm)/DPEPO doped with 6 wt% of **7** (20 nm)/DPEPO (10 nm)/2,2',2”-(1,3,5-benzinetriyl)-tris(1-phenyl-1-H-benzimidazole) (TPBi, 30 nm)/LiF (0.8 nm)/Al (100 nm) (Cui et al., 2017). The electroluminescence spectrum showed good agreement with the PL spectrum of **7** in a DPEPO host. The CIE coordinates were (0.16, 0.08). The maximum external quantum efficiency (EQE_max_) was 2.0%. Considering the low PLQY of **7** (15%), the EQE_max_ value would have been 0.75% if **7** had acted as a conventional fluorescence emitter. The obtained EQE_max_ clearly indicated that **7** acted as a TADF emitter in this OLED. Therefore, the homo-junction design has potential in the development of deep blue TADF materials.

## Conclusions

In summary, we prepared conceptual TADF materials based on a homo-junction design analyzed via a combinatorial computational method. The obtained TADF material with the homo-junction design showed deep blue emission when incorporated into an OLED. The results of NMR experiments identified a mechanism for reducing the Δ*E*_ST_ in the homo-junction design, that is, electron back donation in an excited state. The electron donation from a weak donor (LUMO-distributed DBF) to a strong donor (HOMO-distributed DMAc) at the ground state provided a small Δ*E*_ST_ without changing its oscillator strength. We demonstrated an expanded kinetics analysis based on four states. The conventional three-state kinetic analysis could not be applied to several TADF materials exhibiting three exponential decays in the solid-state emission. In contrast, rate constants provided by the four-state analysis could explain the results very well. Further discussion on the meaning of a “T_n_ state” in actual photophysical processes is necessary. For example, the T_n_ state maybe an actual high energy triplet state but maybe electronically non-relaxed state of T_1_ by surrounding molecules. However, the results clearly suggested that the highly emissive TADF materials required large rate constants for radiative decay, spin flip and endothermic IC in the solid state. This indicated that the formation of a finely tuned HOMO-LUMO overlap, large spin-orbit coupling and small conformational changes were mandatory in the excited state. The TADF materials obtained in this study had limited PLQYs at 300 K. To obtain highly emissive TADF materials with the homo-junction design, a large oscillator strength material with a small Δ*E*_ST_ will likely be necessary. On-going screening by combinatorial DFT calculation should lead to promising blue TADF materials.

## Materials and Methods

### Chemicals and Instruments

All reactants and solvents were used as purchased from Tokyo Chemical Industry (Tokyo, Japan) or Fuji Film-Wako Chemicals (Tokyo, Japan). All reactions were carried out under N_2_ atmosphere. In general, the evaporation and concentration of solvents were carried out under reduced pressure below 50°C, unless otherwise noted. Proton nuclear magnetic resonance (^1^H NMR) spectra were obtained using a Biospin Avance III 500 spectrometer (Bruker, MA, USA) with THF-*d*_8_ as the solvent. Peak multiplicities are given as: s, singlet; d, doublet; dd, double doublet; ddd, double double doublet; t, triplet; dt, double triplet. Mass spectra were measured in positive-ion atmospheric pressure solid analysis probe (ASAP) mode using a Waters 3100 mass detector (Waters, MA, USA). Absorption spectra of the samples were measured using an ultraviolet-visible-near infrared spectrometer (Lambda 950-PKA, Perkin-Elmer, MA, USA). The photoluminescence quantum yield (PLQY) was measured using a PLQY measurement system (Quantaurus-QY, Hamamatsu Photonics, Hamamatsu, Japan). The transient photoluminescence (PL) decay characteristics of samples were measured using an emission lifetime measurement system (Quantaurus-Tau, Hamamatsu Photonics, Hamamatsu, Japan). The transient PL emission and PL decay of **1**–**5** in mCBP films was recorded under vacuum conditions by a streak camera (C4334, Hamamatsu Photonics, Hamamatsu, Japan) with a nitrogen laser (337 nm, 20 Hz, Ken-X, Usho Optical System, Japan) as an excitation source. The nitrogen laser was employed to measure prompt emission lifetimes.

### Combinatorial DFT Calculations

The 87 donor units used to construct TADF molecules were analyzed by DFT calculation with the B3LYP/6-31+g^*^ (Lee et al., 1988; Becke, 1993) level of theory on Jaguar 9.0 software package (Bochevarov et al., 2013; Jaguar 9.0, 2016; Schrödinger LLC 2015). Because our target was blue TADF emitters, units estimated to have S_1_ and T_1_ energy levels <2.5 eV were excluded from the donor list. _S_D-_W_D combinations and their initial structures for DFT calculation were generated by using these donor units with the MM2 level on MacroModel 11.0 software package (MacroModel 11.0, 2015; Schrödinger LLC 2015). Generated _S_D-_W_D combinations were screened by the DFT calculation with the B3LYP/6-31+g^*^ level of theory using Jaguar. By checking with the HOMO-LUMO distribution, the _S_D-_W_D combinations were selected as a first screening for TADF candidates. Then, candidates having Δ*E*_ST_ <0.3 eV were also screened using the M06-2X/6-31+g^*^ level of theory (Zhao and Truhlar, 2008), and these candidates were then verified both in vertical and adiabatic excited state energies by DFT calculation with the LC-ωPBE/6-31+g^*^ (Vydrov and Scuseria, 2006) level of theory using Gaussian16 (Frisch et al., 2016; Gaussian Inc 2016). The NTO analysis of sD-wD combinations also performed with the B3LYP/6-31+g^*^ level of theory using Jaguar.

### Photophysical Measurement

Synthesized DMAc-DBF compounds **1**–**7** were measured in inert gas saturated toluene solution (1.0 × 10^−5^ mol L^−1^) or thermally evaporated film (6 wt% in DPEPO) under vacuum condition.

### Synthesis of DMAc-DBF Compounds

Compounds **1**–**7** were synthesized by the Buchwald-Hartwig amination with 9,9-dimethyl acridine and the corresponding brominated dibenzofuran. The synthesis **1** is given below as a representative procedure.

### General Synthesis

A mixture of 9,9-dimethyl acridine (0.42 g, 2 mmol), 1-bromodibenzofuran (0.24 g, 1 mmol), tri-tert-butylphosphonium tetrafluoroborate (35 mg, 0.12 mmol), sodium *tert*-butoxide (0.23 g, 2.4 mmol), and tris(dibenzylideneacetone)dipalladium (0) (35 mg, 0.06 mmol) was dissolved in anhydrous toluene (40 mL) under an inert atmosphere. After refluxing overnight, the reaction mixture was washed with water and the organic phase was separated, dried with sodium sulfate, filtered to remove sodium sulfate, and then reduced by rotary evaporation. The residue was purified by silica-gel column chromatography (Wako gel 60, eluent was chloroform: hexane = 1: 6). The target compound **1** was obtained as a white powder. The obtained material was purified by sublimation twice. Compounds **2**–**7** were synthesized similarly using the corresponding brominated dibenzofuran.

**10-(dibenzofuran-1-yl)-9,9-dimethylacridine (1)**: Yield, 60%; ^1^H NMR (500 MHz, THF-d_8_): δ 7.84 (d, 1H, *J* = 8.0 Hz, H^4^ on dibenzofuran (DBF)), 7.76 (t, 1H, *J* = 8.0 Hz, H^3^ on DBF), 7.61 (d, 1H, *J* = 8.0 Hz, H^6^ on DBF), 7.58 (dd, 2H, *J* = 1.0, 7.5 Hz, H^4^ on dimethylacridane (DMAc)), 7.39 (ddd, 1H, *J* = 1.0, 7.5, 8.0 Hz, H^7^ on DBF), 7.35 (d, 1H, *J* = 8.0 Hz, H^2^ on DBF), 7.22 (dd, 1H, *J* = 1.0, 7.5 Hz, H^9^ on DBF), 7.02 (d, 1H, *J* = 7.5 Hz, H^8^ on DBF), 6.88 (dt, 2H, *J* = 1.0, 8.0 Hz, H^3^ on DMAc), 6.80 (dt, 2H, *J* = 1.0, 7.5 Hz, H^2^ on DMAc), 6.15 (dd, 2H, *J* = 1.0, 8.0 Hz, H^4^ on DMAc), 1.97 (s, 3H, Me on DMAc), 1.68 (s, 3H, Me on DMAc) ppm; MS (ASAP) m/z 376.15 [M+H]^+^.

**10-(dibenzofuran-2-yl)-9,9-dimethylacridine (2)**: Yield, 90%; ^1^H NMR (500 MHz, THF-d_8_): δ 8.06 (d, 1H, *J* = 2.0 Hz, H^1^ on DBF), 8.03 (dd, 1H, *J* = 1.0, 7.5 Hz, H^9^ on DBF), 7.87 (d, 1H, *J* = 8.5 Hz, H^4^ on DBF), 7.66 (d, 1H, *J* = 8.0 Hz, H^6^ on DBF), 7.52 (ddd, 1H, *J* = 1.0, 7.5, 8.0 Hz, H^7^ on DBF), 7.46 (dd, 2H, *J* = 2.0, 7.0 Hz, H^1^ on DMAc), 7.43 (dd, 1H, *J* = 2.0, 8.5 Hz, H^3^ on DBF), 7.36 (t, 1H, *J* = 7.5 Hz, H^8^ on DBF), 6.87 (dt, 2H, *J* = 2.0, 7.5 Hz, H^3^ on DMAc), 6.85 (dt, 2H, *J* = 1.5, 7.5 Hz, H^2^ on DMAc), 6.26 (dd, 2H, *J* = 1.5, 7.5 Hz, H^4^ on DMAc), 1.69 (s, 6H, Me on DMAc) ppm; MS (ASAP) m/z 376.32 [M+H]^+^.

**10-(dibenzofuran-3-yl)-9,9-dimethylacridine (3)**: Yield, 63%; ^1^H NMR (500 MHz, THF-d_8_): δ 8.34 (d, 1H, *J* = 8.0 Hz, H^1^ on DBF), 8.16 (dd, 1H, *J* = 1.0, 7.5 Hz, H^9^ on DBF), 7.69 (d, 1H, *J* = 8.0 Hz, H^6^ on DBF), 7.68 (d, 1H, *J* = 1.5 Hz, H^4^ on DBF), 7.56 (ddd, 1H, *J* = 1.0, 7.5, 8.0 Hz, H^7^ on DBF), 7.50 (dd, 2H, *J* = 1.5, 7.5 Hz, H^1^ on DMAc), 7.45 (t, 1H, *J* = 7.5 Hz, H^8^ on DBF), 7.36 (dd, 1H, *J* = 1.5, 8.0 Hz, H^2^ on DBF), 6.92 (dt, 2H, *J* = 1.5, 7.5 Hz, H^3^ on DMAc), 6.90 (dt, 2H, *J* = 1.5, 7.5 Hz, H^2^ on DMAc), 6.31 (dd, 2H, *J* = 1.5, 7.5 Hz, H^4^ on DMAc), 1.72 (s, 6H, Me on DMAc) ppm; MS (ASAP) m/z 376.11 [M+H]^+^.

**10-(dibenzofuran-4-yl)-9,9-dimethylacridine (4)**: Yield, 75%; ^1^H NMR (500 MHz, THF-d_8_): δ 8.25 (dd, 1H, *J* = 1.0, 8.0 Hz, H^1^ on DBF), 8.14 (dd, 1H, *J* = 1.5, 7.5 Hz, H^9^ on DBF), 7.64 (t, 1H, *J* = 8.0 Hz, H^2^ on DBF), 7.54 (dd, 1H, *J* = 1.0, 8.0 Hz, H^3^ on DBF), 7.53 (dd, 2H, *J* = 2.0, 7.0 Hz, H^1^ on DMAc), 7.50 (dd, 1H, *J* = 1.5, 8.0 Hz, H^6^ on DBF), 7.47 (dt, 1H, *J* = 1.5, 8.0 Hz, H^7^ on DBF), 7.40 (ddd, 1H, *J* = 1.5, 7.5, 8.0 Hz, H^8^ on DBF), 6.91 (dt, 2H, *J* = 2.0, 7.0 Hz, H^3^ on DMAc), 6.88 (dt, 2H, *J* = 1.5, 7.0 Hz, H^2^ on DMAc), 6.26 (dd, 2H, *J* = 1.5, 7.0 Hz, H^1^ on DMAc), 1.72 (s, 6H, Me on DMAc) ppm; MS (ASAP) m/z 376.10 [M+H]^+^.

**2,8-bis(9,9-dimethylacridin-10-yl)dibenzofuran (5)**: Yield, 86%; ^1^H NMR (500 MHz, THF-d_8_): δ 8.13 (d, 1H, *J* = 2.0 Hz, H^1^ on DBF), 8.00 (d, 1H, *J* = 8.5 Hz, H^4^ on DBF), 7.55 (t, 1H, *J* = 2.0, 8.5 Hz, H^3^ on DBF), 7.48 (dd, 2H, *J* = 1.5, 7.5 Hz, H^1^ on DMAc), 6.92 (dt, 2H, *J* = 1.5, 7.5 Hz, H^3^ on DMAc), 6.88 (dt, 2H, *J* = 1.5, 7.5 Hz, H^2^ on DMAc), 6.32 (dd, 2H, *J* = 1.5, 7.5 Hz, H^4^ on DMAc), 1.70 (s, 6H, Me on DMAc) ppm; MS (ASAP) m/z 583.54 [M+H]^+^.

**3,7-bis(9,9-dimethylacridin-10-yl)dibenzofuran (6)**: Yield, 55%; ^1^H NMR (500 MHz, THF-d_8_): δ 8.46 (d, 1H, *J* = 8.5 Hz, H^1^ on DBF), 7.77 (d, 1H, *J* = 1.5 Hz, H^4^ on DBF), 7.52 (dd, 2H, *J* = 1.5, 7.5 Hz, H^1^ on DMAc), 7.45 (t, 1H, *J* = 1.5, 8.5 Hz, H^2^ on DBF), 6.95 (ddd, 2H, *J* = 1.5, 7.5, 8.0 Hz, H^3^ on DMAc), 6.92 (dt, 2H, *J* = 1.5, 7.5 Hz, H^2^ on DMAc), 6.35 (dd, 2H, *J* = 1.5, 8.0 Hz, H^4^ on DMAc), 1.74 (s, 6H, Me on DMAc) ppm; MS (ASAP) m/z 583.39 [M+H]^+^.

**4,6-bis(9,9-dimethylacridin-10-yl)dibenzofuran (7)**: Yield, 88%; ^1^H NMR (500 MHz, THF-d_8_): δ 8.31 (dd, 1H, *J* = 1.0, 7.5 Hz, H^1^ on DBF), 7.66 (d, 1H, *J* = 7.5 Hz, H^2^ on DBF), 7.58 (t, 1H, *J* = 1.0, 7.5 Hz, H^3^ on DBF), 7.31 (dd, 2H, *J* = 1.5, 7.5 Hz, H^1^ on DMAc), 6.76 (dt, 2H, *J* = 1.5, 8.0 Hz, H^3^ on DMAc), 6.70 (ddd, 2H, *J* = 1.0, 7.5, 8.0 Hz, H^2^ on DMAc), 6.19 (dd, 2H, *J* = 1.0, 8.0 Hz, H^4^ on DMAc), 1.42 (s, 6H, Me on DMAc) ppm; MS (ASAP) m/z 583.26 [M+H]^+^.

## Data Availability Statement

All datasets generated for this study are included in the article/Supplementary Material.

## Author Contributions

YT performed some experiments, most analysis, construction of rate equations, and co-wrote the paper. KT performed most organic synthesis, most experiment, and some analysis. KI, TM, HK, and MH performed molecular modeling and combinatorial DFT calculation. FB helped construction of rate equations. YG performed some organic synthesis, and some experiment. HN and CA interpreted the results, and co-wrote the paper.

## Conflict of Interest

TM, HK, and MH are employed by the company Schrödinger Inc. The remaining authors declare that the research was conducted in the absence of any commercial or financial relationships that could be construed as a potential conflict of interest.
