# Supplementary material for: Molecular Design Based on Donor-Weak Donor Scaffold for Blue Thermally-Activated Delayed Fluorescence Designed by Combinatorial DFT Calculations
Source: Front Chem. 2020 May 6;8:403. doi: 10.3389/fchem.2020.00403 (PMC7218164; doi:10.3389/fchem.2020.00403)
Supplement: Supplementary file 1 [file Data_Sheet_1.pdf]

Supplementary Material

Molecular Design Based on Donor-Weak Donor Scaffold for Blue Thermally-Activated Delayed Fluorescence Designed by Combinatorial DFT Calculations

Youichi Tsuchiya[1,2*], Keita Tsuji[1,3], Ko Inada[1,2], Fatima Bencheikh[1,2], Yan Geng[1], Thomas J. L. Mustard[4], H. Shaun Kwak[4], Mathew D. Halls[4], Hajime Nakanotani[1,2,3], Chihaya Adachi[1,2,3,5*]

[1]*Center for Organic Photonics and Electronics Research (OPERA), Kyushu University, 744, Motooka, Nishi-ku, Fukuoka 819-0395, Japan*
[2]*Japan Science and Technology Agency (JST), ERATO, Adachi Molecular Exciton Engineering Project, Fukuoka 819-0395, Japan*
[3]*Department of Chemistry and Biochemistry, Kyushu University. 744, Motooka, Nishi-ku, Fukuoka 819-0395, Japan.*
[4]*Schrödinger Inc, 10201 Wateridge Circle, Suite 220, San Diego, California, 92121, USA*
[5]*International Institute for Carbon Neutral Energy Research (WPI-I2CNER), Kyushu University, Fukuoka 819-0395, Japan*

*** Correspondence:**
Chihaya Adachi adachi@cstf.kyushu-u.ac.jp
Youichi Tsuchiya tsuchiya@opera.kyushu-u.ac.jp

Contents:

1. Estimation of k_r^S, f and Q from absorption and emission spectra
2. Detailed derivation of expanded kinetic equations
3. Supplementary Figures and Tables

References

1 Estimation of k_r^S, f and Q from absorption and emission spectra (Hirata et al., 2015)

The relationship between the radiative decay rate (k_r^S) and the absorption coefficient (ε) in fluorescent molecules can be expressed as

$$k_r^S = 2.88 \times 10^{-9} n^2 \langle \bar{\nu}_f{}^{-3} \rangle^{-1} \int \varepsilon(\nu_a) \mathrm{d} \ln \nu_a \qquad \text{(S1)}$$

$$\langle \bar{\nu}_f{}^{-3} \rangle^{-1} = \frac{\int f(\nu_f) \mathrm{d}\nu_f}{\int f(\nu_f) \nu_f{}^{-3} \mathrm{d}\nu_f} \qquad \text{(S2)}$$

where ν_f is the fluorescence wavenumber, $f(\nu_f)$ is the fluorescence spectrum at ν_f, ν_a is the absorption wavenumber, $\varepsilon(\nu_a)$ is the coefficient at ν_a, and n is the refractive index of the solvent at the average wavenumber of fluorescence $\langle \nu_f \rangle$ (Strickler and Berg, 1962). To obtain $\langle \bar{\nu}_f{}^{-3} \rangle^{-1}$, two curves were plotted for each spectrum. One curve was the normalized intensities by emission peak maxima at each frequency, and the other curve was $\bar{\nu}_f{}^{-3}$ times this value at each frequency. The ratio of the areas under the curves gives $\langle \bar{\nu}_f{}^{-3} \rangle^{-1}$. In an analogous process, $\langle \nu_f \rangle$ is given by

$$\langle \nu_f \rangle = \frac{\int f(\nu_f) \nu_f \mathrm{d}\nu_f}{\int f(\nu_f) \mathrm{d}\nu_f} \qquad \text{(S3)}$$

$\int \varepsilon(\nu_a) \mathrm{d} \ln \nu_a$ is the area under the curve of $\varepsilon(\nu_a)$ for the charge transfer (CT) absorption band (red solid curves in Figure S2) at $\ln \nu_a$. The relationship between the oscillator strength for absorption (f), transition dipole moment for absorption (Q), and $\varepsilon(\nu_a)$ can be experimentally determined as

$$f = 4.32 \times 10^{-9} n^{-1} \int \varepsilon(\nu_a) \mathrm{d}\nu_a = \left(\frac{8\pi^2 m_e c \langle \nu_f \rangle}{3he^2} \right) |Q|^2 \qquad \text{(S4)}$$

where m_e is the electron mass, c is the speed of light, h is Planck's constant, and e is the elementary charge (Mulliken, 1939). Here, n is the refractive index of the solvent at the peak maximum of the gauss curve of CT absorption.

2 Detailed derivation of expanded kinetic equations

When the direct non-radiative decay processes from the singlet (S_1) and higher triplet (T_n) state to ground state (S_0) are scarcely occur and all intersystem crossing passes through the T_n state, the efficiency of each process (Φ) can written by

$$\begin{aligned} \Phi_{FL} + \Phi_{ISC} &= \Phi_{FL} + \Phi_{ISC}(\Phi_{RISC} + \Phi_{IC}^T) = \Phi_{FL} + \Phi_{DE1} + \Phi_{ISC}\Phi_{IC}^T(\Phi_{RIC}^T + \Phi_r^T + \Phi_{nr}^T) \\ &= \Phi_{FL} + \Phi_{DE1} + \Phi_{ISC}\Phi_{IC}^T\Phi_{RIC}^T + \Phi_{ISC}\Phi_{IC}^T\Phi_r^T + \Phi_{ISC}\Phi_{IC}^T\Phi_{nr}^T \\ &= \Phi_{FL} + \Phi_{DE1} + \Phi_{DF2} + \Phi_{Phos} + \Phi_{ISC}\Phi_{IC}^T\Phi_{nr}^T = \Phi_{FL} + \Phi_{DE1} + \Phi_{DE2} + \Phi_{ISC}\Phi_{IC}^T\Phi_{nr}^T \\ &= \Phi_{PLQY} + \Phi_{ISC}\Phi_{IC}^T\Phi_{nr}^T = 1 \qquad \text{(S5)} \end{aligned}$$

where Φ_{FL} is the fraction of radiative decay from the excited lowest energy singlet (S_1) state (fluorescence), Φ_{ISC} is the fraction of intersystem crossing (ISC), Φ_{RISC} is the fraction of reverse intersystem crossing (RISC) of triplet excitons, Φ_{IC}^{T} is the fraction of exothermic internal conversion (IC) from the T_n state to the lowest triplet (T_1) state, Φ_{RIC}^{T} is the fraction of endothermic reverse IC from the T_1 to T_n state, Φ_{r}^{T} is the fraction of radiative decay of triplet excitons from the T_1 state, Φ_{nr}^{T} is the fraction of non-radiative decay of triplet excitons from the T_1 state, Φ_{DE1} is the fraction of delayed emission provided by $\Phi_{ISC}\Phi_{RISC}$ (i.e. faster delayed fluorescence, Φ_{DF1}), Φ_{DF2} is the fraction of latter delayed fluorescence provided by $\Phi_{ISC}\Phi_{IC}^{T}\Phi_{RIC}^{T}$, Φ_{Phos} is the fraction of phosphorescence provided by $\Phi_{ISC}\Phi_{IC}^{T}\Phi_{r}^{T}$, Φ_{DE2} is the total fraction of delayed emission *via* the T_1 state ($\Phi_{DF2} + \Phi_{Phos}$), and Φ_{PLQY} is the fraction of total emission efficiency provided by $\Phi_{FL} + \Phi_{DE1} + \Phi_{DE2}$.

The singlet exciton lifetime (τ_S) and each triplet exciton lifetime (τ_{Tn} and τ_{T1}) are written by

$$\tau_S = \tau_{FL} = \frac{1}{k_r^S + k_{ISC}} \qquad \text{(S6)}$$

$$\tau_{Tn} = \tau_{DE1} = \frac{1}{\Phi_{FL}k_{RISC} + k_{IC}^{T}} \qquad \text{(S7)}$$

$$\tau_{T1} = \tau_{DE2} = \tau_{DF2} = \tau_{Phos} = \frac{1}{\Phi_{RISC}k_{RIC}^{T} + k_r^T + k_{nr}^T} \qquad \text{(S8)}$$

where k_r^S is the radiative decay rate from the S_1 state, k_{ISC} is the ISC rate, k_{nr}^S is the non-radiative decay rate from the S_1 state, k_{RISC} is the RISC rate, k_{IC}^T is the exothermic IC rate to the T_1 state, k_{RIC}^T is the endothermic reverse IC rate from the T_1 state, k_r^T is the radiative decay rate from the T_1 state and k_{nr}^T is the non-radiative decay rate from the T_1 state. Considering the ISC/RISC cycle (limited by k_{RISC}) and the IC/RIC cycle (limited by k_{RIC}), the actual decay speed through the RISC and IC processes should depend on $\Phi_{FL} + \Phi_{nr}^S = 1 - \Phi_{ISC}$ and Φ_{RISC}, respectively. τ_S, τ_{Tn} and τ_{T1} are the lifetimes of the observed fluorescence (τ_{FL}), first component of delayed emission (τ_{DE1}) and second component of delayed emission (τ_{DE2}), respectively.

The singlet and each triplet decay rate (k^S, k_1^T and k_2^T) are written by

$$k^S = k_r^S + k_{ISC} = 1/\tau_{FL} \qquad \text{(S9)}$$

$$k_1^T = \Phi_{FL}k_{RISC} + k_{IC}^T = 1/\tau_{DE1} \qquad \text{(S10)}$$

$$k_2^T = \Phi_{RISC}k_{RIC}^T + k_r^T + k_{nr}^T = 1/\tau_{DE2} \qquad \text{(S11)}$$

Summarizing the above discussion, each efficiency is written by

$$\Phi_{FL} = \frac{k_r^S}{k_r^S + k_{ISC}} = k_r^S\tau_{FL} \qquad \text{(S12)}$$

$$\Phi_{ISC} = \frac{k_{ISC}}{k_r^S + k_{ISC}} = k_{ISC}\tau_{FL} \qquad \text{(S13)}$$

$$\Phi_{RISC} = \frac{\Phi_{FL}k_{RISC}}{\Phi_{FL}k_{RISC} + k_{IC}^T} = (1 - \Phi_{ISC})k_{RISC}\tau_{DE1} \qquad \text{(S14)}$$

$$\Phi_{IC}^T = \frac{k_{IC}^T}{\Phi_{FL}k_{RISC} + k_{IC}^T} = k_{IC}^T\tau_{DE1} = 1 - \Phi_{RISC} \qquad \text{(S15)}$$

$$\Phi_{RIC}^T = \frac{\Phi_{RISC}k_{RIC}^T}{\Phi_{RISC}k_{RIC}^T + k_r^T + k_{nr}^T} = \Phi_{RISC}k_{RIC}^T\tau_{DE2} \qquad \text{(S16)}$$

$$\Phi_r^T = \frac{k_r^T}{\Phi_{RISC}k_{RIC}^T + k_r^T + k_{nr}^T} = k_r^T\tau_{DE2} \qquad \text{(S17)}$$

$$\Phi_{nr}^T = \frac{k_{nr}^T}{\Phi_{RISC}k_{RIC}^T + k_r^T + k_{nr}^T} = k_{nr}^T\tau_{DE2} = \frac{1 - \Phi_{PLQY}}{\Phi_{ISC}\Phi_{IC}^T} \qquad \text{(S18)}$$

Summarizing the above equations, each rate constant is written by

$$k_r^S = \frac{\Phi_{FL}}{\tau_{FL}} = k^S\Phi_{FL} \qquad \text{(S19)}$$

$$k_{ISC} = \frac{\Phi_{ISC}}{\tau_{FL}} = k^S\Phi_{ISC} = k^S(1 - \Phi_{FL}) \qquad \text{(S20)}$$

$$k_{RISC} = \frac{\Phi_{RISC}}{\Phi_{FL}\tau_{DE1}} = \frac{\Phi_{DE1}}{\Phi_{FL}k_{ISC}\tau_{FL}\tau_{DE1}} = \frac{\Phi_{DE1}}{\Phi_{FL}} \cdot \frac{k^S k_1^T}{k_{ISC}} \qquad \text{(S21)}$$

$$k_{IC}^T = \frac{1}{\tau_{DE1}} - \Phi_{FL}k_{RISC} = k_1^T - \Phi_{FL}k_{RISC} \qquad \text{(S22)}$$

$$k_{RIC}^T = \frac{\Phi_{RIC}^T}{\tau_{DE2}\Phi_{RISC}} = k_2^T \cdot \frac{\Phi_{RIC}^T}{\Phi_{RISC}} \approx k_2^T \cdot \frac{\Phi_{DE2}}{\Phi_{ISC}\Phi_{RISC}\Phi_{IC}^T} \quad (\because \Phi_r^T \approx 0) \qquad \text{(S23)}$$

$$k_r^T = \frac{\Phi_r^T}{\tau_{DE2}} = k_2^T\Phi_r^T \qquad \text{(S24)}$$

$$k_{nr}^T = \frac{\Phi_{nr}^T}{\tau_{DE2}} = k_2^T\Phi_{nr}^T = k_2^T \cdot \frac{1 - \Phi_{PLQY}}{(1 - \Phi_{FL})\Phi_{IC}^T} \qquad \text{(S25)}$$

When we use the assumption of Φ_r^T as 0, the k_{RIC}^T is overestimated. From Eq. S5, the relationship of Φ_{RIC}^T and Φ_r^T is written by

$$\Phi_{RIC}^T + \Phi_r^T = \frac{\Phi_{DE2}}{\Phi_{ISC}\Phi_{IC}^T} \qquad \text{(S28)}$$

When the phosphorescence ratio in the second delayed emission (Φ_{DE2}) can be estimated, both Φ_{RIC}^T and Φ_r^T can be determined by Eq. S28. It would be reasonable to assume Φ_r^T as 0 at 300 K, while the phosphorescence ratio can be obtained by fitting of the prompt fluorescence emission and cryogenic phosphorescence emission.

3 Supplementary Figures and Tables

Table S1. Calculated and estimated energy levels of pre-synthesized homo-junction materials.

Structure		HOMO distribution[a]	LUMO Distribution[a]	Functional1//Functional2	Vertical[b] S_1 (eV)	Vertical[b] T_1 (eV)	Vertical[b] ΔE_{ST} (eV)	Adiabatic[c] S_1 (eV)	Adiabatic[c] T_1 (eV)	Adiabatic[c] ΔE_{ST} (eV)	PLQY (%)[f]	Lifetime (ns)[f]
**S1**	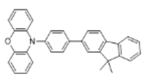	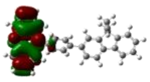	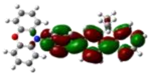	B3LYP//B3LYP	2.87	2.86	0.01	-	-	-		
				M06-2X//M06-2X	3.80	3.31	0.49	3.42	2.33	0.73	16	72
				LC-ωPBE*[d]//B3LYP	3.51	2.94	0.57	3.14	2.15	0.67	(22)	(129)
				Experimental[e]	-	-	-	3.02	2.64	0.38		
**S2**	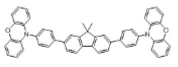	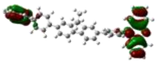	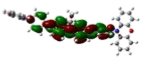	B3LYP//B3LYP	2.75	2.74	0.01	-	-	-		
				M06-2X//M06-2X	3.90	3.33	0.57	3.11	2.28	0.55	19	120
				LC-ωPBE*[d]//B3LYP	3.37	2.92	0.45	2.88	2.10	0.55	(25)	(120)
				Experimental[e]	-	-	-	2.95	2.48	0.47		
**S3**	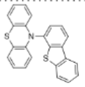	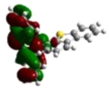	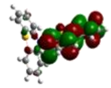	B3LYP//B3LYP	2.75	2.73	0.03	-	-	-		
				M06-2X//M06-2X	3.66	3.18	0.48	-	-	-	0.9	3.0/ 7.9
				LC-ωPBE*[d]//B3LYP	3.68	3.21	0.47	2.88	1.91	0.56	(1.9)	(3.6/ 13.1)
				Experimental[e]	-	-	-	3.08	2.65	0.43		
**S4**	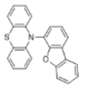	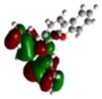	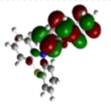	B3LYP//B3LYP	3.05	3.04	0.01	-	-	-		
				M06-2X//M06-2X	3.99	3.52	0.47	-	-	-	1.4	2.7
				LC-ωPBE*[d]//B3LYP	3.62	3.16	0.46	2.89	1.89	0.56	(1.9)	(3.0/ 10.3)
				Experimental[e]	-	-	-	3.07	2.67	0.40		

[a] Calculated using the B3LYP/6-31G(d) level of theory. [b] Vertical values were estimated using the PCM(toluene)-TDA-Functional1/6-31+g(d)//Functional2/6-31g(d) level of theory. [c] Adiabatic values were estimated using the PCM(toluene)-TDA-Functional1/6-31+g(d)//PCM(toluene)-TD(singlet)/DFT(triplet)-CAM-B3LYP/6-31g(d) level of theory. [d] ω values were tuned for **S1**, **S2**, **S3** and **S4** as 0.1831, 0.1713, 0.1868 and 0.1908, respectively. [e] Experimental values were measured in toluene (1.0×10^{-5} mol L^{-1}). [f] PLQY and lifetime values were measured in toluene (1.0×10^{-5} mol L^{-1}) under air saturated or N_2 saturated (bracketed) conditions.

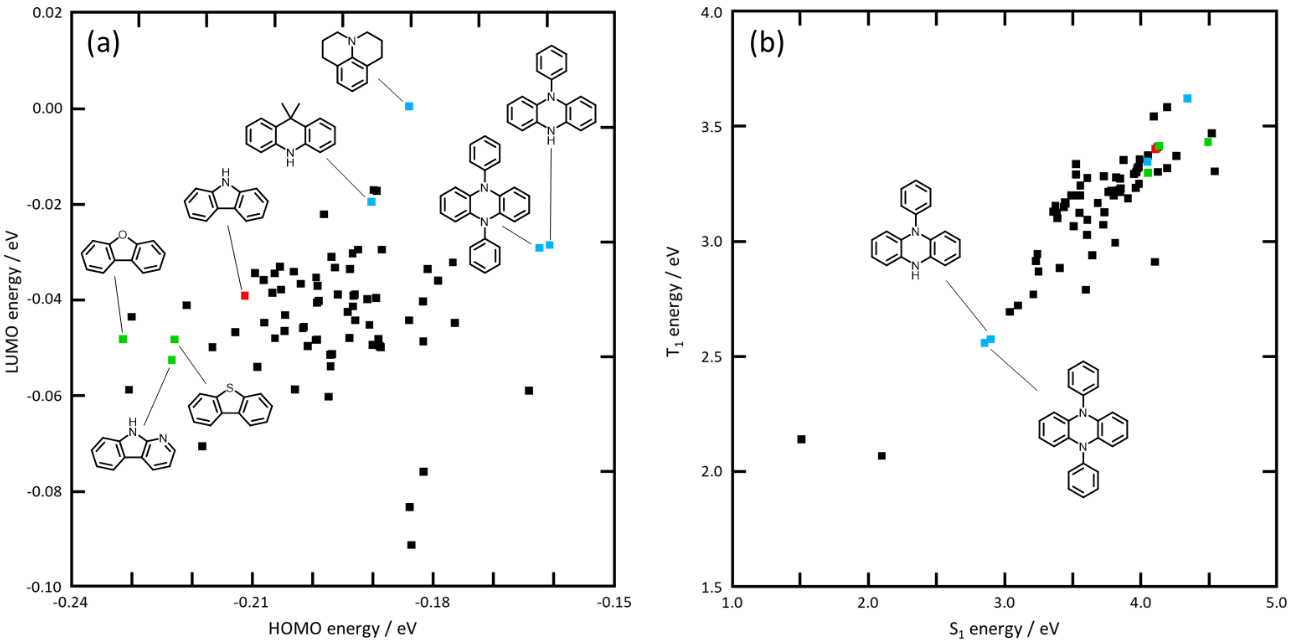

Figure S1. (a) HOMO/LUMO level and (b) S_1/T_1 level plots of donor units using combinatorial B3LYP DFT calculations.

Table S2. DFT calculation results for final TADF candidates of **1**–**7**.

TADF candidates	Functional1//Functional2	Vertical[a]			Adiabatic[b]		
		S_1 (ev)	T_1 (ev)	ΔE_{ST} (eV)	S_1 (ev)	T_1 (ev)	ΔE_{ST} (eV)
**1**	B3LYP//B3LYP	3.07	3.05	0.02	3.64	2.63	0.02
	M06-2X//M06-2X	3.88	3.78	0.10	3.45	3.29	0.12
	LC-ωPBE*[c]//B3LYP	3.64	3.42	0.22	3.30	2.76	0.32
**2**	B3LYP//B3LYP	3.13	3.08	0.05	N/A[d]	N/A[d]	N/A[d]
	M06-2X//M06-2X	4.04	3.73	0.31	3.86	3.54	0.33
	LC-ωPBE*[c]//B3LYP	3.70	3.39	0.31	3.54	3.21	0.33
**3**	B3LYP//B3LYP	3.04	3.02	0.02	2.67	2.65	0.01
	M06-2X//M06-2X	3.84	3.74	0.11	3.47	3.26	0.13
	LC-ωPBE*[c]//B3LYP	3.39	3.61	0.22	3.31	2.74	0.33
**4**	B3LYP//B3LYP	3.08	3.07	0.01	2.68	2.66	0.01
	M06-2X//M06-2X	3.85	3.78	0.07	3.44	3.27	0.08
	LC-ωPBE*[c]//B3LYP	3.50	3.41	0.09	3.19	2.78	0.19
**5**	B3LYP//B3LYP	3.03	2.98	0.05	N/A[d]	N/A[d]	N/A[d]
	M06-2X//M06-2X	3.95	3.73	0.22	3.79	3.50	0.29
	LC-ωPBE*[c]//B3LYP	3.62	3.38	0.24	3.43	3.17	0.26
**6**	B3LYP//B3LYP	3.00	2.97	0.03	N/A[d]	N/A[d]	N/A[d]
	M06-2X//M06-2X	3.94	3.75	0.19	3.71	3.56	0.15
	LC-ωPBE*[c]//B3LYP	3.57	3.39	0.18	3.43	3.26	0.17
**7**	B3LYP//B3LYP	2.99	2.97	0.02	N/A[d]	N/A[d]	N/A[d]
	M06-2X//M06-2X	3.87	3.61	0.26	3.68	3.42	0.26
	LC-ωPBE*[c]//B3LYP	3.55	3.44	0.11	3.37	3.13	0.24

[a] Vertical values were estimated using the PCM(toluene)-TDA-Functional1/6-31+g(d)//Functional2 /6-31g(d) level of theory. [b] Adiabatic values were estimated using the PCM(toluene)-TDA-Functional1/6-31+g(d)//PCM(toluene)-TD(singlet)/DFT(triplet)-CAM-B3LYP/6-31+g(d) level of theory. [c] ω values were tuned for **1**, **2**, **3**, **4**, **5**, **6** as 0.1850, 0.1843, 0.1839, 0.1841, 0.1843, 0.1843 and 0.1843, respectively. [d] Adiabatic T_1 energies from B3LYP single-point calculations were not available since TDA calculations at B3LYP failed to converge at a triplet state that is lower in energy than S_1 state at the geometry optimized with CAM-B3LYP.

Table S3. HOMO-LUMO distributions and natural transition orbitals (NTO) for the lowest singlet excitation of **1**–**7** calculated by DFT with the B3LYP/6-31+g(d) level of theory (Martin, 2003).

	Structure	HOMO distribution	LUMO distribution	NTO (S_1) hole	NTO (S_1) particle
**1**	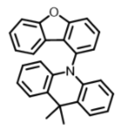	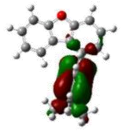	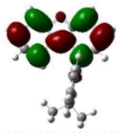	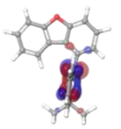	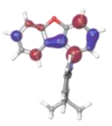
**2**	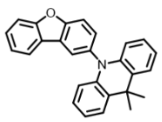	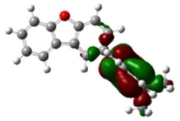	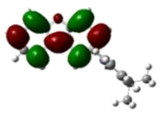	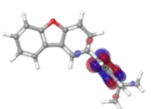	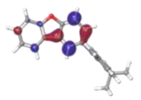
**3**	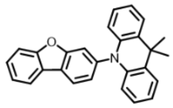	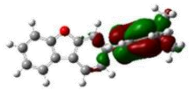	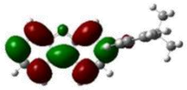	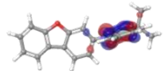	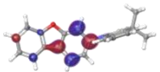
**4**	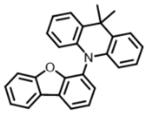	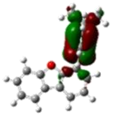	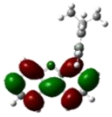	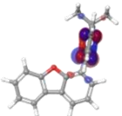	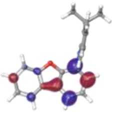
**5**	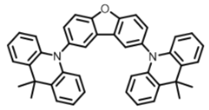	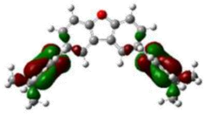	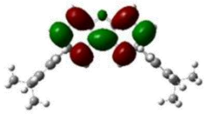	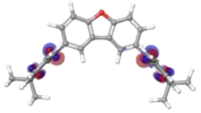	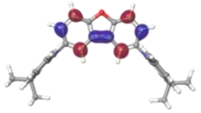
**6**	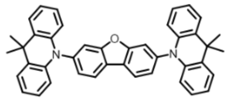	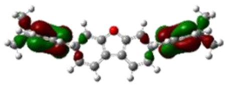	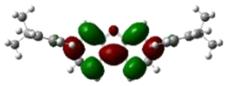	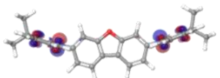	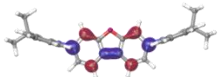
**7**	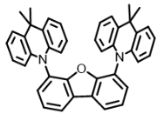	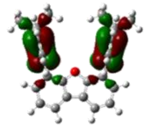	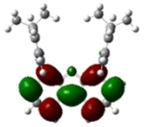	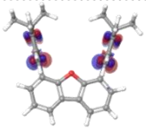	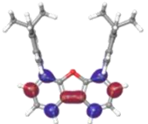

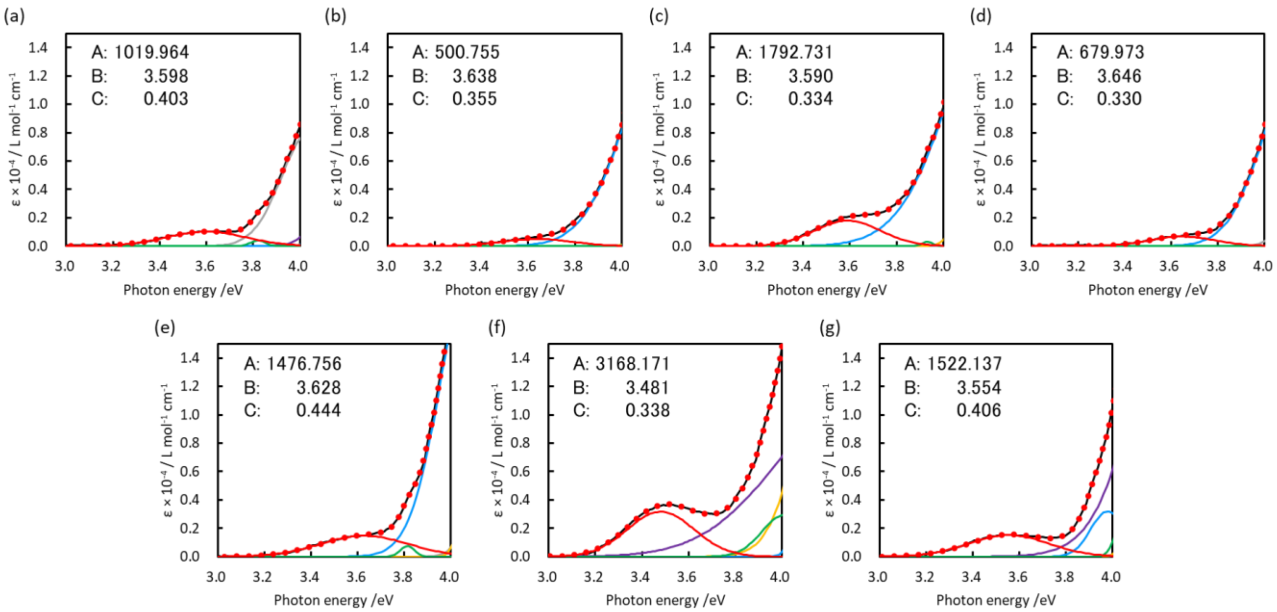

Figure S2. Absorption characteristics of guests in toluene solution; (a) **1**, (b) **2**, (c) **3**, (d) **4**, (e) **5**, (f) **6**, (g) **7**. In general, the lines represent the molar absorption coefficient (ε) *vs.* absorption photon energy characteristics. Colored lines represent fitting curves when the fine spectra (dotted red line) are expressed by a summation of each curve based on the Gaussian model: $\varepsilon_n(v) = A_n \exp(-(v-B_n)^2/2C_n^2)$ (n = 1, 2, 3…), where An is the amplitude, B_n is the average wavenumber and C_n is the distribution parameter. Solid black lines are experimental spectra. The absorption with the lowest absorption wavenumber (red solid lines) corresponds to CT absorption in each of **1**–**7**.

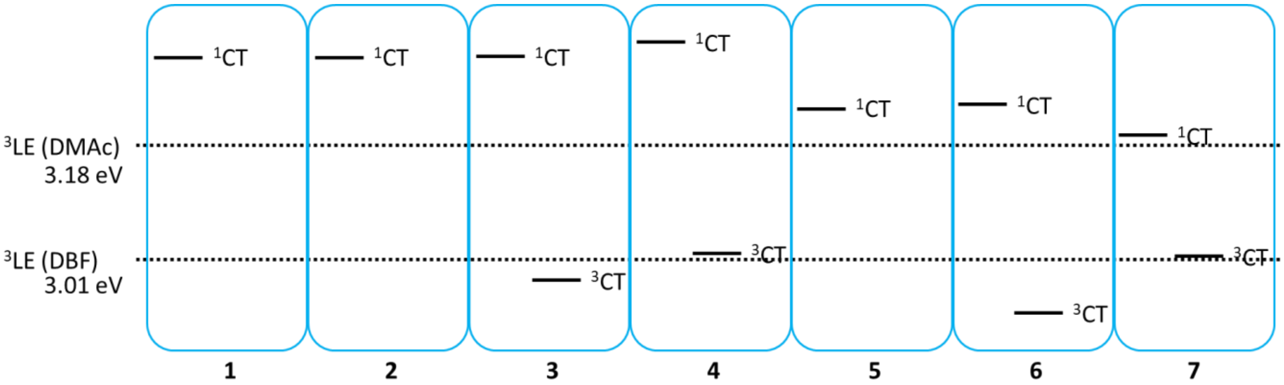

Figure S3. Summary of observed exciton energy levels of charge transfer singlet state (^{1}CT), charge transfer triplet state (^{3}CT) and local excited triplet state (^{3}LE) of **1**-**7**. ^{3}CT for **1**, **2**, and **5** could not be observed by spectroscopy, ^{3}CT for **4** and **7** were observed in phosphorescence as a mixing to ^{3}LE.

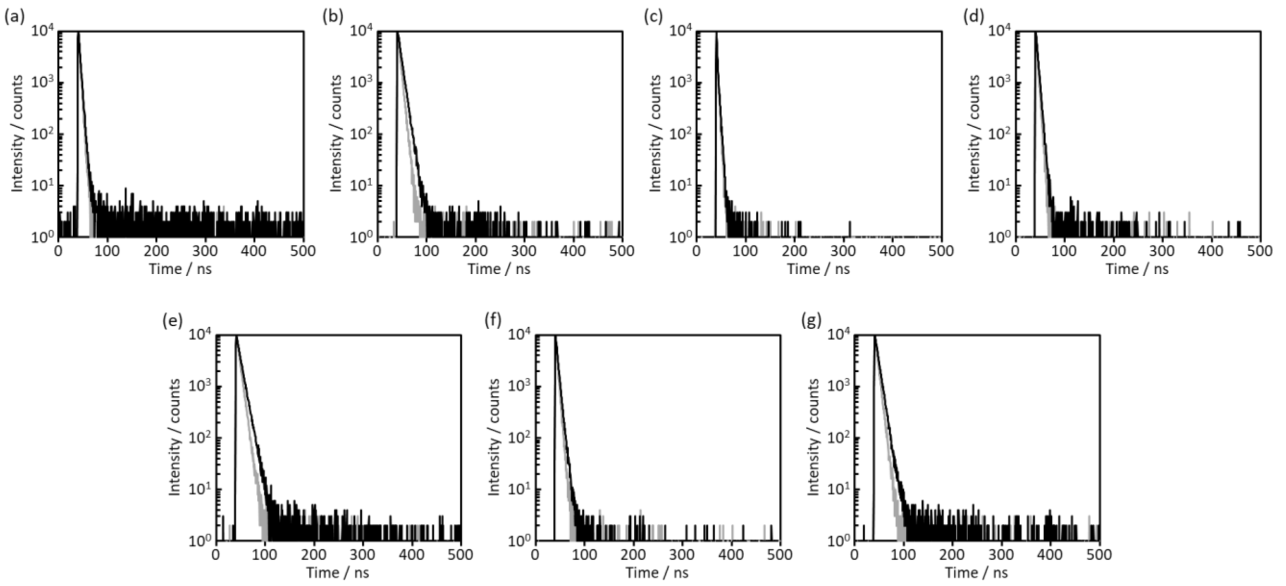

Figure S4. Transient emission decay curves in toluene solution (1.0×10^{-5} mol L^{-1}); (a) **1**, (b) **2**, (c) **3**, (d) **4**, (e) **5**, (f) **6**, (g) **7**; black lines, N_2 saturated conditions; gray lines, air saturated conditions.

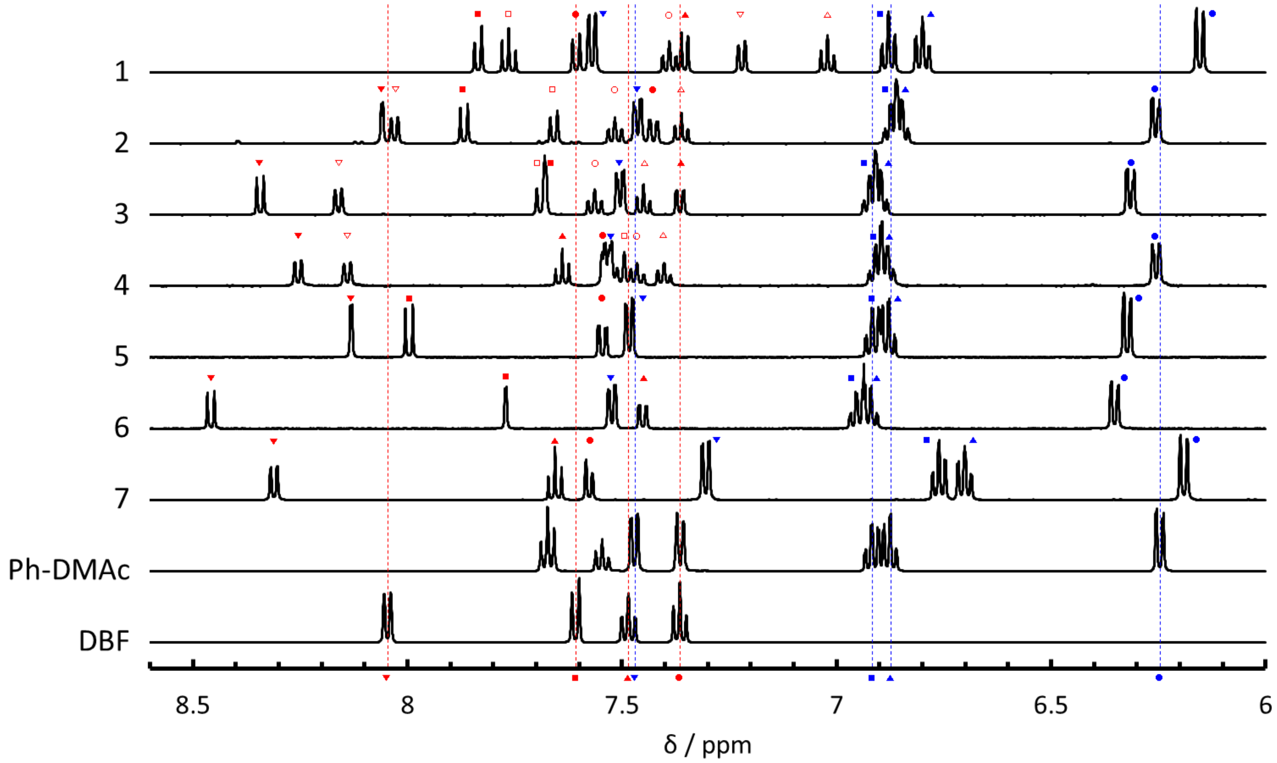

Figure S5. ^{1}H NMR spectra of **1**–**7**, **Ph-DMAc** and **DBF** in THF-d_8 solution. Broken red and blue lines indicate proton peaks based on **DBF** and DMAc units of **Ph-DMAc**, respectively.

Table S4. ^{1}H NMR chemical shifts (ppm) of DMAc units for **1**–**7** and **Ph-DMAc** in THF-d_8

	H^1	H^2	H^3	H^4	Me
**1**	7.58	6.80	6.88	6.15	1.97/ 1.68
**2**	7.46	6.85	6.87	6.26	1.69
**3**	7.50	6.90	6.92	6.31	1.72
**4**	7.53	6.88	6.91	6.26	1.72
**5**	7.48	6.88	6.92	6.32	1.70
**6**	7.52	6.92	6.95	6.35	1.74
**7**	7.31	6.70	6.76	6.19	1.42
**Ph-DMAc**	7.47	6.88	6.92	6.25	1.68

Table S5. ^{1}H NMR chemical shifts (ppm) of DBF units for **1**–**7** and DBF in THF-d_8.

	H^1 (H^9)	H^2 (H^8)	H^3 (H^7)	H^4 (H^6)
**1**	-	7.35	7.76	7.84
	(7.22)	(7.02)	(7.39)	(7.61)
**2**	8.06	-	7.43	7.87
	(8.03)	(7.36)	(7.52)	(7.66)
**3**	8.34	7.36	-	7.68
	(8.16)	(7.45)	(7.56)	(7.69)
**4**	8.25	7.64	7.54	-
	(8.14)	(7.40)	(7.47)	(7.50)
**5**	8.13	-	7.55	8.00
**6**	8.46	7.45	-	7.77
**7**	8.31	7.66	7.58	-
**DBF**	8.05	7.37	7.48	7.61

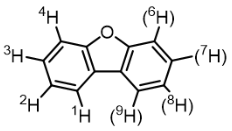

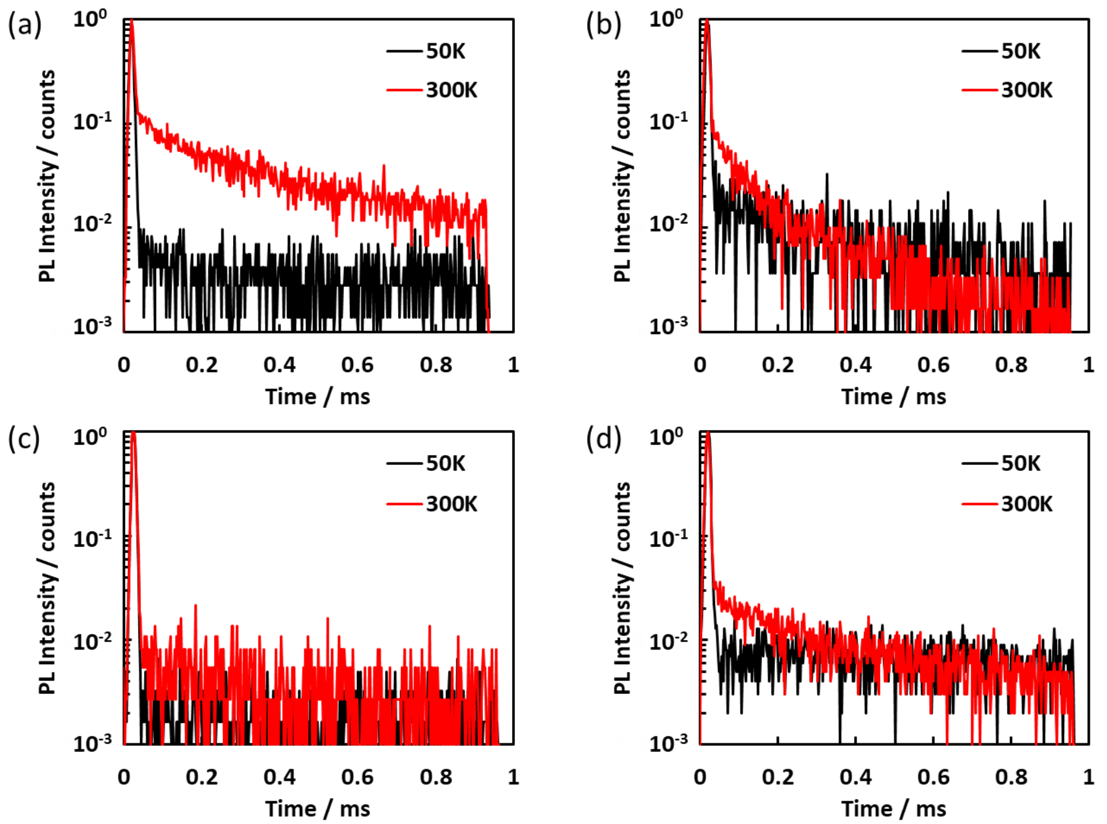

Figure S6. Temperature dependency of transient emission decay of **DPEPO** films doped with (a) **5** and (b) **6** (6 wt%).

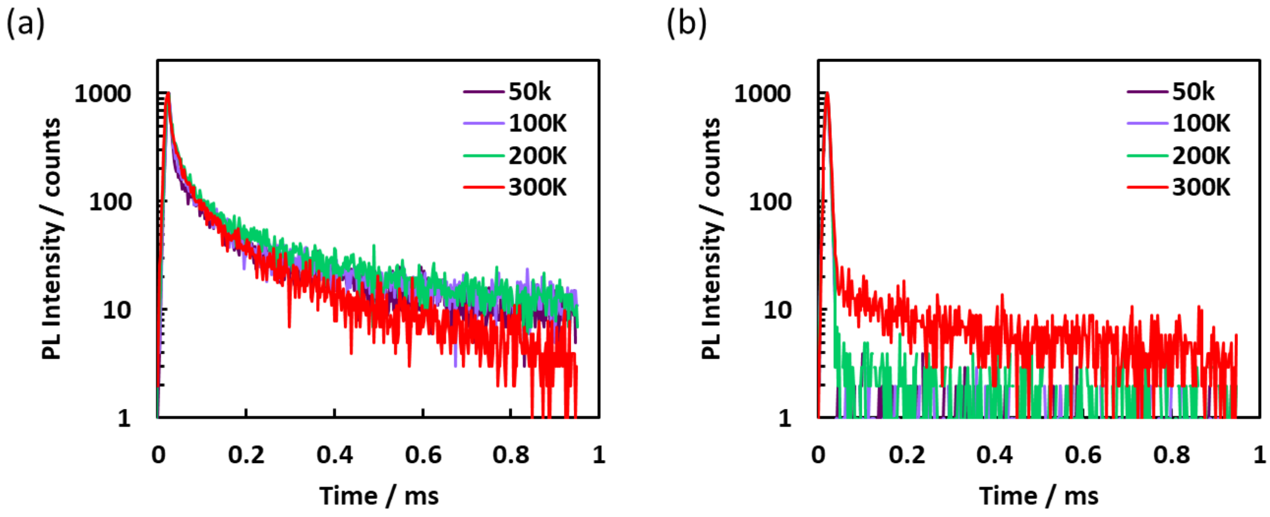

Figure S7. Temperature dependency of transient emission decay of **DPEPO** films doped with (a) **1**, (b) **2**, (c) **3** and (d) **6** (6 wt%).

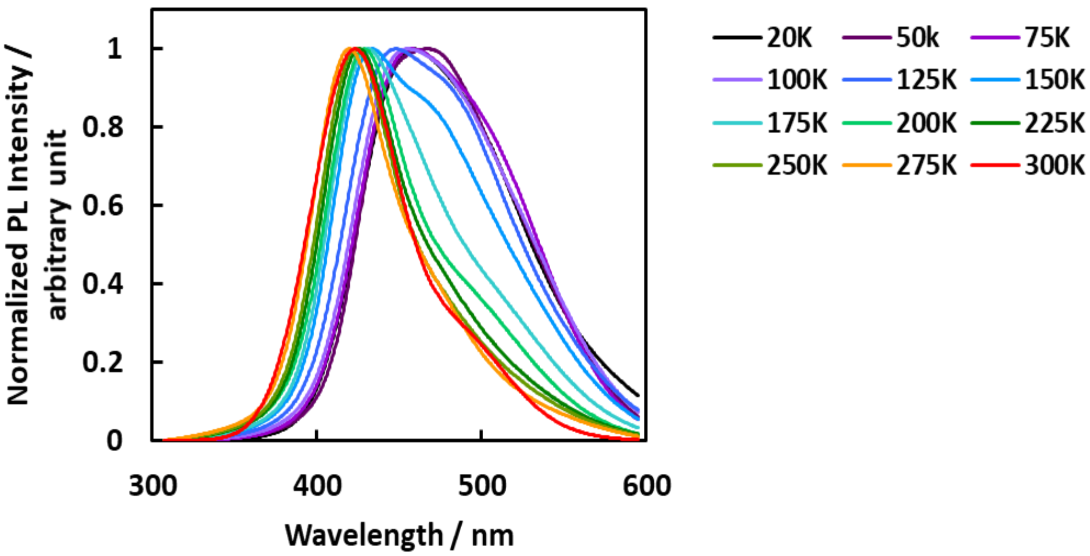

Figure S8. Temperature dependency of delayed emission spectra of **DPEPO** films doped with **7** (6 wt%).

Table S6. Photophysical properties using the four-state analysis for **1**–**7**; Φ_r^T were assumed as 0 for simplicity.

	**1**	**2**	**3**	**4**	**5**	**6**	**7**
Φ_{PLQY}	0.15	0.14	0.14	0.11	0.27	0.39	0.15
S_1 (eV)	3.38	3.43	3.42	3.41	3.18	3.25	3.37
T_1 (eV)	3.08	3.07	3.00	3.18	3.10	2.90	3.08
ΔE_{ST} (eV)	0.30	0.36	0.42	0.23	0.08	0.35	0.29
τ_{prompt} (ns)	4.1	6.8	3.6	3.6	3.0	3.5	3.4
τ_{delay1} (μs)	231	78	8.13	237	63	212	172
τ_{delay2} (ms)	3.7	0.53	-	15.6	0.43	7.3	2.2
Φ_{prompt}	0.012	0.044	0.020	0.007	0.051	0.051	0.036
Φ_{delay1}	0.035	0.043	0.120	0.006	0.107	0.019	0.043
Φ_{delay2}	0.103	0.053	-	0.097	0.112	0.319	0.072
k^S_r (10^7 s^{-1})	0.30	0.64	0.55	0.20	1.69	1.46	1.05
k_{ISC} (10^8 s^{-1})	2.41	1.41	2.72	2.76	3.16	2.71	2.84
k_{RISC} (10^4 s^{-1})	1.25	1.33	0.08	0.38	3.51	0.19	0.72
k^T_{IC} (10^3 s^{-1})	4.18	12.2	-	4.20	14.0	4.62	5.56
k^T_{RIC} (10^2 s^{-1})	8.25	24.2	-	9.86	27.8	22.8	8.00
k^T_{nr} (10^2 s^{-1})	2.43	1.79	1.08	0.58	20.3	0.89	4.18

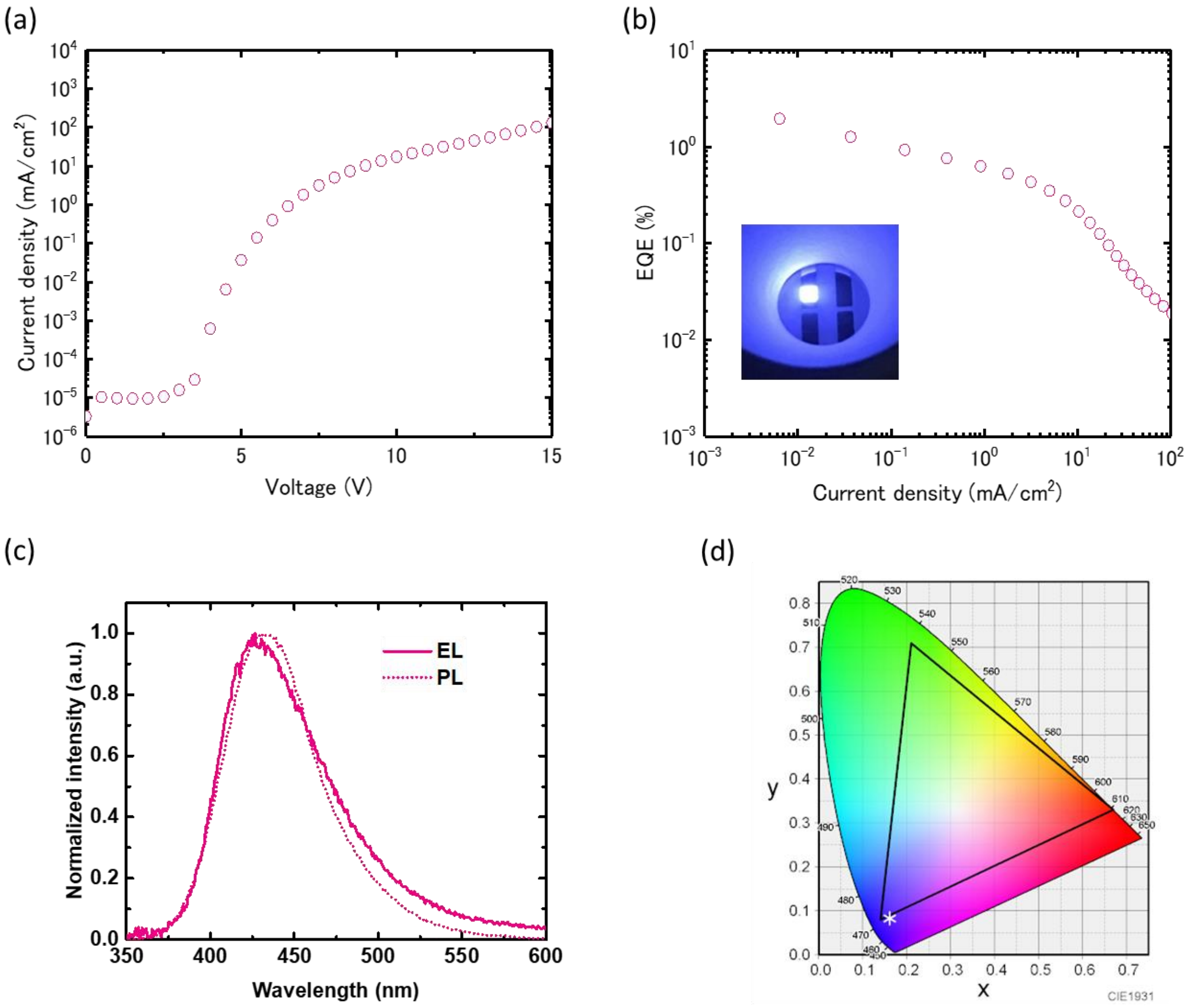

Figure S9. Device performance of **7**; (a) current density-voltage (*J*-*V*) plot, (b) external quantum efficiency (EQE)-luminance plot, (c) electroluminescence (EL) and PL spectra and (d) location of EL emission on the CIE coordinate system (0.161, 0.083).

References

Hirata S, Sakai Y, Masui K, Tanaka H, Lee S-Y, Nomura H, Nakamura N, Yasumatsu M, Nakanotani H, Zhang Q, Shizu K, Miyazaki H, Adachi C. Highly efficient blue electroluminescence based on thermally activated delayed fluorescence. *Nat. Mater*. (2015) 14: 330−336.

Martin R.L. Natural transition orbitals. *J. Chem. Phys.* (2003) 118: 4775−4777.

Mulliken R S. Intensities of Electronic Transitions in Molecular Spectra I. Introduction. *J. Chem. Phys.* (1939) 7: 14−20.

Strickler S J, Berg R A. Relationship between Absorption Intensity and Fluorescence Lifetime of Molecules. *J. Chem. Phys.* (1962) 37: 814−822.
